# Form and function of archaeal genomes

**DOI:** 10.1042/BST20221396

**Published:** 2022-12-13

**Authors:** Stephen D. Bell

**Affiliations:** 1 Molecular and Cellular Biochemistry Department, Indiana University, Bloomington, IN 47405, U.S.A.; 2 Biology Department, Indiana University, Bloomington, IN 47405, U.S.A.

**Keywords:** archaea, CID, cohesin, condensin, SMC, TAD

## Abstract

A key maxim in modernist architecture is that ‘form follows function'. While modernist buildings are hopefully the product of intelligent design, the architectures of chromosomes have been sculpted by the forces of evolution over many thousands of generations. In the following, I will describe recent advances in our understanding of chromosome architecture in the archaeal domain of life. Although much remains to be learned about the mechanistic details of archaeal chromosome organization, some general principles have emerged. At the 10–100 kb level, archaeal chromosomes have a conserved local organization reminiscent of bacterial genomes. In contrast, lineage-specific innovations appear to have imposed distinct large-scale architectural features. The ultimate functions of genomes are to store and to express genetic information. Gene expression profiles have been shown to influence chromosome architecture, thus their form follows function. However, local changes to chromosome conformation can also influence gene expression and therefore, in these instances, function follows form.

## General features of archaeal genomes

The seminal studies of Woese and Fox revealed Archaea to be a distinct domain of life from Bacteria and Eukaryotes [1]. More recent advances in phylogenetic methods, coupled with massively increased taxonomic sampling have revealed deep divisions between archaeal phyla and have provided support for the emergence of eukaryotes from within the archaeal domain of life [2]. Metagenomic studies have provided a wealth of primary sequence information on archaeal species yet comparatively few organisms have been successfully grown as pure cultures, and, of these cultivated species, only a handful have proven amenable to genetic manipulation. Much of what we currently know about archaeal molecular genetics and chromosome biology is therefore derived from a limited number of species, most notably, members of the *Sulfolobales* order of the crenarchaea and the *Halobacteriales* and *Thermococcales* orders of the euryarchaea.

Thus far, all archaeal genomes are encoded on circular chromosomes and, like bacterial genomes, encode polycistronic units. As in bacteria, it is believed that transcription and translation are coupled in archaea [3]. Echoing the deep phyletic divide between cren- and euryarchaea, there are some fundamental differences in cell cycle logic and DNA replication mode between *Sulfolobus* and the euryarchaeal *Haloferax* and *Thermococcus* (Table 1). *Sulfolobus* species, and other crenarchaea, have a cell cycle in which a new-born cell has a single copy of its chromosome [4]. After a brief G1 period DNA replication initiates. *Sulfolobus* chromosomes possesses three replication origins, of which two (*oriC1* and *oriC3*) fire at the same time thereby defining the start of S-phase and the third origin fires a few minutes into S-phase [5]. Ultimately, all cells fire all three replication origins. Following the completion of S-phase, approximately half the cell cycle is spent with two copies of the chromosome and fluorescence *in situ* hybridization (FISH) of a limited number of loci around the genome provided evidence for the persistence of sister chromatid cohesion for the majority of the G2 period [6]. This cohesion correlated with the existence of hemi-catenane junctions between the daughter molecules. At the end of G2, genomes compact concomitant with their segregation, in a process that likely involves *Sulfolobus* homologs of a bacterial ParA protein, termed SegA and its DNA binding partner, SegB [7]. *Sulfolobus* cells then divide utilizing the ESCRT machinery [8,9].

**Table 1:** Chromosomal vital statistics of representative archaeal strains

Species	Phylum	Ploidy	Genome size (Mb)	Replication origin number	SMC proteins	Compartments	CIDs	Loops
*Thermococcus kodakarensis* KOD1	Euryarchaea	∼20	2.09	0–1^1^	SMC Rad50	No	Yes	Yes
*Haloferax volcanii* DS2	Euryarchaea	∼20^2^	2.85^3^ (main) 0.085 (pHV1) 0.438 (pHV3) 0.636 (pHV4)	3^3^ 1 1 1	Sph1 Sph2 Sph3 Sph4 Rad50	No	Yes	Yes
*Sulfolobus islandicus* REY15A	Crenarchaea	1C–2C	2.52	3	ClsN RAD50	Yes, A/B	Yes	Yes

1 The single replication origin of *Thermococcus* is dispensable for growth under laboratory conditions [14];

2 Figures for copy number of the main chromosome The ploidy of *H. volcanii* is strongly influenced by growth conditions — cells grown in phosphate-limiting medium have two copies of the main chromosome whereas cell grown under phosphate-rich conditions have up to 40 copies [49];

3 The karyotype of *H. volcanii* is remarkably plastic with different laboratory strains derived from the reference strain DS2 having undergone fusion or excision of replicons with or from the main chromosome [13,50].

In contrast with the tightly organized 1C–2C ploidy control and regimented cell cycle of *Sulfolobus*, the euryarchaea are typically polyploid [10]. For example, *Haloferax volcanii* possesses ∼20 copies of its main chromosome. Studies in synchronized halophilic archaea that followed BrdU incorporation to monitor DNA replication or utilized GFP fused to the single-stranded DNA binding protein, provided evidence to suggest that DNA replication occurs throughout the cell cycle with no defined pre- and post-replicative phases. Indeed, replication was even observed in cells undergoing division. Quantitation of the SSB-GFP foci indicated that only 1–3 chromosomes replicate at a given time (for a recent review see [11]). Thus, for the halophiles, at least in regard to replication, there is a non-equivalence between chromosomes. Whether all chromosomes in a given cell have the same transcription profile and/or have the same conformation are currently unresolved issues. Another intriguing difference between the model euryarchaea and the crenarchaeote *Sulfolobus* lies in DNA replication mode. *Sulfolobus* has three DNA replication origins and the activity of at least one is required for viability [12]. In contrast, while *Haloferax volcanii* typically uses DNA replication origins during normal growth, it is possible to delete all active origins and retain viability [13]. Remarkably, origin-lacking cells even double more rapidly than their wild-type counterparts under laboratory growth conditions. Replication in these cells is likely mediated via homologous recombination as the cells are highly sensitive to levels of the RadA protein, the ortholog of bacterial RecA and eukaryotic Rad51 [13]. In *Thermococcus kodakarensis*, studies in exponentially growing cells failed to provide any evidence for replication origin firing or defined replication initiation sites [14]. However, genetic studies implicated origin function in surviving prolonged starvation. It was suggested that during nutrient deprivation, ongoing cell division could eventually reduce the cells to single chromosome content. In a 1C state, with no homologs present, replication origin function, rather than homologous recombination, would presumably be required to facilitate the restart of chromosome replication.

## Conserved architectural features of archaeal chromosomes

Our understanding of the conformation of bacterial chromosomes has been established by a combination of microscopy approaches, using FISH, fluorescent repressor/operator systems (FROS), a FROS-related technology utilizing an orthogonal fluorescent-ParB/*parS* system and through the application of proximity ligation chromosome conformation capture (3C)-based methodologies [15]. While FISH has been employed on a limited number of loci in *Sulfolobus*, it has not been extensively used in archaea. Nor have FROS-like technologies been employed in any archaeal species to date. Archaeal chromosome conformation studies have therefore focused on the application of 3C technologies to archaeal cultures [16–19]. The results of these studies of asynchronous populations report on the dominant and persistent architectural features. A further caveat must be borne in mind with the polyploid euryarchaea, as it is possible that the various copies of the chromosome have distinct and non-equivalent architectures [11]. In *Sulfolobus*, the existence of sister chromatid cohesion during the majority of the G2 phase of the cell cycle gives some confidence that 3C methods do indeed capture the conformation of both chromosomes in a given cell [6]. A clear goal for future work will be to apply 3C methods to single archaeal cells. It will also be of considerable interest to perform 3C on synchronized cultures to determine the modulation of chromosome architecture during the course of the cell cycle.

High-resolution 3C studies on both euryarchaeal and crenarchaeal species have revealed the presence of self-interacting domains throughout the genomes [17,18]. The size of these domains range from 25–570 kb in *Haloferax volcanii* and 14–196 kb in *Sulfolobus* species. In both species there is a statistically significant correlation between the location of domain boundaries and highly expressed genes. This combination of scale and boundary definition suggests these structures are analogous to ‘chromosomal interaction domains' (CIDs) first characterized in the bacterium *Caulobacter crescentus* and which are now known to be a universal feature of bacterial chromosome organization [20,21]. Studies in *Sulfolobus* confirmed that, as in bacteria, gene expression plays an active role is establishing boundaries [18]. Global inhibition of transcription using the DNA intercalator Actinomycin D reduced boundary definition and genetic approaches to either delete or insert highly transcribed genes resulted in loss or gain of CID boundaries, respectively. It has been proposed that highly expressed genes may act as CID boundaries by serving as barriers to the diffusion of plectonemic supercoiling in bacteria. It seems highly plausible that a similar mechanism may be at play in archaeal chromosomes. It is worthwhile emphasizing that this proposed dependence of CIDs on a fundamental topological feature of DNA readily accounts for the similarities in CIDs across a broad range of organisms both within and across domains of life even though their transcription machineries and chromatin compaction systems are highly distinct.

In addition to CIDs, the high-resolution studies have provided support for the existence of defined loop structures between disparate loci in archaeal chromosomes. In both *Sulfolobus* and *Haloferax* ∼70 loops of varying length were detected throughout the genome [17,18]. Notably, loops could encompass multiple CIDs, suggesting CIDs and loops are distinct organizational features of the chromosome. Analysis of the identity of the genes in the vicinity of loop anchors in *Sulfolobus* revealed a significant enrichment of genes for protein and RNA components of the ribosome [18]. While the 3C method employed reports on binary interactions, the observation that, within the population, a single anchor point could interact with a number of discrete loci suggests that a hub-like organization could exist, allowing the spatial colocalization of genes involved in ribosome biogenesis. In both *Haloferax* and *Sulfolobus*, loops appear to be dependent upon transcription, as Actinomycin D treatment led to a loss of loops in both species [17,18]. Additionally, entry into stationary phase reduced loops, and, in *Sulfolobus*, the ‘ribosomal gene’ loops were particularly impacted. The mechanistic basis of loop formation is currently not fully resolved. One explanation could be long-range bridging events between transcription factors bound at the anchor loci. Another possibility, at least for the *Sulfolobus* ribosomal loops, is that coordinated translation and ribosomal assembly could provide an attractive force that creates a nexus for ribosome biogenesis. Finally, as will be discussed below, in *Haloferax*, deletion of a gene for a SMC-superfamily protein had an impact on loop formation, albeit less of an effect than observed upon Actinomycin D treatment or entry into stationary phase [17].

## Compartmentalization of *Sulfolobus* chromosomes

While loops and CIDs are shared features of the crenarchaea and euryarchaeal studied thus far, work in *Sulfolobus* has revealed an additional architectural level, compartmentalization that, thus far, has not been observed in euryarchaeal species [16]. In addition to the ∼100 kb CIDs, *Sulfolobus* chromosomes possess an additional level of larger scale self-interacting segments. These segments form discrete higher-order interactions, generating a checker board-like plaid pattern in the Hi-C contact maps. The strong bias of these interactions defines two distinct compartments. Strikingly, this compartmentalized architecture has also been observed in mammalian cells, where the compartments are referred to as A and B type [22]. The same A/B nomenclature has been adopted for *Sulfolobus*. As with the mammalian A compartment, the *Sulfolobus* A compartment possesses the majority of the highly expressed genes in the genome. The B compartment in *Sulfolobus* is more lowly expressed, contains non-essential genes and also is enriched for transposable elements [16]. Thus, at the functional level, the *Sulfolobus* A and B compartments are reminiscent of the phenomena of euchromatin and heterochromatin, respectively, in eukaryotes. It is important to stress that while there is a conceptual and functional analogy, the mechanistic bases of definition of the A/B compartments in *Sulfolobus* and of eukaryotic euchromatin and heterochromatin are entirely distinct.

Notably, the three replication origins in the *Sulfolobus* chromosome are located in the A compartment. However, inactivation of the origins by deletion of the cognate initiator protein gene did not affect compartmentalization. Thus, ongoing replication is not required for the maintenance of A compartment identity [16]. However, as discussed below, it seems likely that over multi-generational, evolutionary timescales the replication program will have contributed to sculpting the conformation of the chromosome. Compartment identity, like CIDs and loops, is dependent on the transcriptional profile of cells. Inhibition of transcription with Actinomycin D abrogated compartments. Entry into stationary phase, during which a relative increase in transcription occurs within the B compartment, also triggers a loss of compartmentalization. Interestingly, disruption of the genes for the extremely abundant cell surface S-layer subunits also resulted in a loss of compartmentalization [18]. This is an extremely pleiotropic mutation with cells exhibiting altered morphology, size and ploidy [23]. However, this observation could perhaps hint at a contribution of cell shape to underlying chromosome organization. It is important to stress that compartmentalization is distinct from CID formation. CIDs are found in both A and B compartment and even under conditions where compartmentalization is lost, CIDs persist. In contrast with the independence of compartments and CIDs, the overall number of loop structures was reduced in conditions where compartments were weakened, suggesting a causal interplay between these two phenomena [18]. Given that 80% of loops are intra-compartmental, one possibility is that loops could act to reinforce the compartmentalized architecture.

## The contributions of SMC-superfamily proteins to archaeal chromosome conformation

SMC proteins are member of an extended family of ATPases, possessing a bipartite ATPase head domain, a coiled-coil domain and, at the opposite end of the coiled-coil from the ATPase domain, a hinge domain that serves as a dimerization module. Depending on the species and protein complex in question, SMC proteins form homo- or heterodimers and further associate with additional partner proteins. For excellent overviews of the phylogeny of the SMC superfamily and the closely related RAD50 family of proteins, the reader is directed to recent articles by Yoshinaga and Inagaki [24,25]. In eukaryotes, the SMC-containing complexes, cohesin and condensin are well documented to play key roles in establishing and maintaining chromosome tertiary structure. While cohesin is a eukaryotic innovation, condensins are near universal, being found in eukaryotes, bacteria and many, but not all, archaea [26]. In bacteria, condensin facilitates juxtaposition of chromosome arms and data support a loop-extrusion model to generate the juxtaposition [27]. While helping to establish long-range architecture in bacteria, there is currently no evidence to indicate a role for bacterial condensins in establishing or maintaining CIDs [28]. In eukaryotes, cohesin functions to establish CID-like domains, the Topologically Associating Domains (TADs). Cohesin is enriched at TAD boundaries and thus a TAD could be viewed as a loop extruded by cohesin [29–31]. In mammals, the oriented DNA binding of the protein CTCF provides the directional cue to limit cohesion movement and thus CTCF and cohesin enrichment are hallmarks of mammalian TAD boundaries [32–34].

While high resolution protein structures have been determined for archaeal condensin components, both for the SMC subunit and the partner proteins ScpA and ScpB [35–37], very little is known about the physiological roles of the archaeal SMC family proteins. Genetic studies of a condensin SMC homolog in the euryarchaeon *Methanococcus voltae* revealed that disruption of the gene resulted in aberrant chromosome segregation and cell shape, engendering a ‘titan cell' phenotype [38]. Another SMC-superfamily member, Sph1, of *Halobacterium salinarum* was implicated in events late in chromosome replication and segregation [39]. More recently, the high-resolution 3C studies in *Haloferax volcanii* tested the contributions of SMC-superfamily proteins to chromosome conformation [17]. The gene for the condensin SMC subunit was non-essential for viability with similar growth rate, morphology and DNA content to wild-type cells. The cells however, displayed a loss of 9 domain boundaries across the genome. Importantly, this was not due to transcriptional changes in the boundary-proximal genes, suggesting a direct architectural role for the protein in boundary definition. Whether this is due to physical localization of the condensin complex at these loci is currently unknown. A modest but significant reduction in loops was also observed in the mutant cells. Deletion mutants of genes for two further SMC-superfamily proteins, RAD50 and Sph4, did not show any significant reduction in boundaries or loops but, like the strain lacking the condensin SMC, showed some reduction in short-range contacts [17].

Condensin is found throughout the archaeal lineages, with the exception of the crenarchaeal phylum, of which *Sulfolobus* is a member. Examination of the genome sequence of *Sulfolobus* identified open-reading frame encoding a signature SMC-related protein that we have termed coalescin, or ClsN [16,40]. ClsN is a small SMC-family protein of 68 kDa with ∼350 amino acids of coiled-coil. Interestingly, the dimerization domain of ClsN appears to be a Zinc-hook motif, a feature previously identified in the RAD50 family of proteins [41].

ChIP-Seq revealed ClsN to be enriched in the B compartment of *Sulfolobus* and over-expression of the *clsN* gene led to a strengthening of compartmentalization with a concomitant further reduction in the already low gene expression in the B compartment. Furthermore, ClsN occupancy, as adjudged by ChIP-Seq, displayed a general anti-correlation with transcription levels of loci with which it associated. Importantly, induction of transcription correlated with loss of ClsN at the induced loci [16]. Thus, there appears to be a mutually antagonistic interplay between gene transcription and coalescin binding. In metazoan, the self-interacting TADs are demarcated by the enrichment of cohesin and the DNA binding protein CTCF at boundaries between TADs. The available data strongly support cohesin acting to extrude loops of DNA with CTCF acting as a polar barrier to cohesin movement [30,31,33,34]. In contrast, in *Sulfolobus* there is no detectable enrichment of coalescin at boundaries between the self-interacting CID domains [18]. Rather, coalescin shows elevated enrichment within the body of B compartment CIDs. Thus, within the B compartment, CIDs seem to be defined by locally high gene expression levels at the boundaries and ClsN enrichment within the CID body. The mechanism of ClsN action is unresolved at this time but the current observations of its localization are compatible with either local dynamic DNA loop extrusion by the protein or a more static binding and second-strand capture models. Many additional aspects of ClsN function remain to be determined: whether dedicated ClsN loaders and unloaders exist, whether ClsN interacts with additional partner proteins and how ClsN localization is modulated during the cell cycle are all key topics requiring resolution.

## Evolutionary implications of compartmentalization

**Figure 1.**
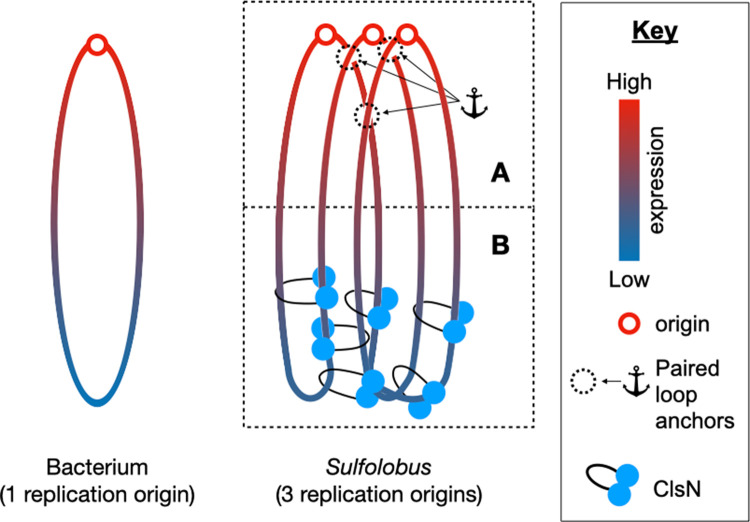
Comparison of a model bacterial chromosome (left) and the chromosome of *Sulfolobus* species (right). Replication origins are shown as red circles. Gene expression levels are indicated by the color gradient from origin-proximal loci with high expression levels (red) to origin distal loci with low expression (blue). Paired loop anchors between co-regulated genes are indicated within dotted circles and binding sites for the *Sulfolobus*-specific SMC superfamily protein ClsN indicated. The *Sulfolobus* A and B compartments are indicated by boxing.

The compartmentalization of *Sulfolobus* chromosomes with its partitioning into more and less transcriptionally active domains prompts comparisons with eukaryotic euchromatin and heterochromatin. However, the basis of the evolution of this modular nature may have mechanistic parallels with bacterial chromosomes. It is well established that many bacterial species show gradients of gene expression from their single DNA replication origins to the termination sites. This is interpreted as a mechanism arising over evolutionary timescales that allows selection for elevated expression of important growth-related genes, in essence by ensuring coupling of gene dosage with early chromosome replication [42]. A similar principle could be at play in *Sulfolobus* with the additional complexity that with three replication origins, three distinct regions can now provide safe harbors for the genes undergoing selection. As alluded to above, looping between regulatory elements that govern coordinate expression of these origin-proximal genes and/or attractive forces mediated by coupled transcription/translation of multi-protein assemblies, such as ribosomes, could provide a mechanism for consolidating the 3D juxtaposition of origin proximal loci. In the case of *Sulfolobus*, the general antagonism between ClsN and transcription results in enrichment of ClsN in transcriptionally quiescent regions. Through loop extrusion and/or second strand capture, ClsN can lead to the coalescence of these less highly expressed genes. Thus, the combination of selection over evolutionary time scales for origin-proximal localization for highly expressed genes, their regulatory looping and the action of ClsN to generate coalesced loci of low transcriptional activity could drive the formation of the compartmentalized structure seen in present-day *Sulfolobus* genomes (Figure 1). It is interesting to note that the definition of compartmentalization [as adjudged by principal component (PC) analyses] is weakest in the vicinity of *oriC2* [19]. It is notable, therefore, that *oriC2* is a *Sulfolobus*-specific replication origin and, presumably, the most recently acquired in this lineage. The heterogeneity in the PC signals in the vicinity of *oriC2* may reflect the ongoing evolutionary streamlining of gene order in the vicinity of this locus.

Finally, the advent of compartmentalization has profound implications for the ongoing evolution of *Sulfolobus* chromosomes. It has been established that *Sulfolobus* chromosomes display uneven rates of single nucleotide polymorphisms (SNPs) [43]. Remarkably, regions of low and high SNP accumulation correspond to the A and B compartments, respectively [19]. Similarly, gene synteny is also more conserved within the A compartment than in the B compartment. Interestingly, proximity to replication termination sites, rather than distance from replication origins, may have a causal role in SNP accumulation in the B compartment [19]. In principle, the different levels of transcription of A and B compartments could contribute to this bias via distinct levels of transcription-coupled repair (TCR) in the two compartments. However, current evidence suggests there is no TCR pathway in *Sulfolobus* [44]. Thus, the mechanistic basis of this differential accumulation of SNPs remains undetermined at this time. It is notable that data derived from Assay for Transposase-Accessible Chromatin (ATAC)-Seq demonstrated that the B compartment is less accessible to transposition events than the A compartment [19]. A key implication of this result is that the B compartment may have a denser, more highly compacted organization than the A compartment. The denser, more restrictive nature of the B compartment may result in slower kinetics of DNA-repair associated events, perhaps explaining the higher mutation rate of this compartment.

Our knowledge of archaeal chromosome architecture and its modulation and regulation is still at a very rudimentary level. Much remains to be determined about the identity and molecular roles of the key players that effect chromosome organization and how these players are regulated throughout the cell-cycle and during culture growth. It is clear that transcription plays a vital role in sculpting the domain organization of archaeal genomes, an illustration of function defining form. Yet, beyond speculation about insulating plectoneme diffusion, how transcriptional activity leads to CID boundaries is unresolved. Indeed, we know essentially nothing about the topological landscape of archaeal chromosomes. While extensive biochemical and structural studies have been performed on archaeal topoisomerases [45], how supercoiling is distributed on archaeal chromosomes and how this distribution could impact on higher-order organization remains unknown. We also know very little at this point about the fine-level chromatin landscape of archaeal cells. A variety of chromatin or nucleoid-associated proteins have been characterized in archaeal species [46]. These include orthologs of histones in essentially all archaeal phyla apart from the crenarchaea. How these various protein interact with, and are distributed on, archaeal genomes and how they contribute to higher order organization remain exciting topics for future work. Understanding the basis of locus-locus loop formation is another key goal. The observation that the evolutionary trajectory of a given locus is influenced by its location in B or A compartment reveals how function can follow form. It is exciting to note that this context-dependent bias in mutation rate is not restricted to archaea, similar epigenome-driven phenomena can be observed in *Arabidopsis* and in human cancer cells [47,48].

## Perspectives

- The position of archaea in the tree of life gives unique insights into the mechanisms and evolution of chromosome organization
- Studies of archaeal chromosome conformation reveal an intriguing blend of features previously associated with bacterial and eukaryotic chromosome organization
- Ongoing studies will reveal the mechanistic basis of chromosome organization via the interplay of DNA topology, gene transcription, chromatin proteins and SMC complexes.

## Abbreviations

CIDs: chromosomal interaction domains
FISH: fluorescence *in situ* hybridization
FROS: fluorescent repressor/operator systems
PC: principal component
SNPs: single nucleotide polymorphisms
TADs: topologically associating domains
TCR: transcription-coupled repair
